# Seizure-Induced Motility of Differentiated Dentate Granule Cells Is Prevented by the Central Reelin Fragment

**DOI:** 10.3389/fncel.2016.00183

**Published:** 2016-07-28

**Authors:** Catarina Orcinha, Gert Münzner, Johannes Gerlach, Antje Kilias, Marie Follo, Ulrich Egert, Carola A. Haas

**Affiliations:** ^1^Experimental Epilepsy Research, Department of Neurosurgery, Medical Center, University of FreiburgFreiburg, Germany; ^2^Faculty of Medicine, University of FreiburgFreiburg, Germany; ^3^Faculty of Biology, University of FreiburgFreiburg, Germany; ^4^Bernstein Center Freiburg, University of FreiburgFreiburg, Germany; ^5^Laboratory for Biomicrotechnology, Department of Microsystems Engineering, University of FreiburgFreiburg, Germany; ^6^Lighthouse Core Facility, Department of Medicine I, Medical Center, University of FreiburgFreiburg, Germany; ^7^BrainLinks-BrainTools, Cluster of Excellence, University of FreiburgFreiburg, Germany

**Keywords:** temporal lobe epilepsy, hippocampus, granule cell dispersion, migration, kainate, motility

## Abstract

Granule cell dispersion (GCD) represents a pathological widening of the granule cell layer in the dentate gyrus and it is frequently observed in patients with mesial temporal lobe epilepsy (MTLE). Recent studies in human MTLE specimens and in animal epilepsy models have shown that a decreased expression and functional inactivation of the extracellular matrix protein Reelin correlates with GCD formation, but causal evidence is still lacking. Here, we used unilateral kainate (KA) injection into the mouse hippocampus, an established MTLE animal model, to precisely map the loss of reelin mRNA-synthesizing neurons in relation to GCD along the septotemporal axis of the epileptic hippocampus. We show that reelin mRNA-producing neurons are mainly lost in the hilus and that this loss precisely correlates with the occurrence of GCD. To monitor GCD formation in real time, we used organotypic hippocampal slice cultures (OHSCs) prepared from mice which express enhanced green fluorescent protein (eGFP) primarily in differentiated dentate granule cells. Using life cell microscopy we observed that increasing doses of KA resulted in an enhanced motility of eGFP-positive granule cells. Moreover, KA treatment of OHSC resulted in a rapid loss of Reelin-producing interneurons mainly in the hilus, as observed *in vivo*. A detailed analysis of the migration behavior of individual eGFP-positive granule cells revealed that the majority of these neurons actively migrate toward the hilar region, where Reelin-producing neurons are lost. Treatment with KA and subsequent addition of the recombinant R3–6 Reelin fragment significantly prevented the movement of eGFP-positive granule cells. Together, these findings suggest that GCD formation is indeed triggered by a loss of Reelin in hilar interneurons.

## Introduction

Characteristic features of mesial temporal lobe epilepsy (MTLE) are recurrent focal seizures and Ammon’s horn sclerosis (AHS) characterized by neuronal loss and granule cell dispersion (GCD), an abnormal broadening of the dentate granule cell layer (GCL; Houser, 1990; Haas et al., 2002). There is recent evidence that a loss of the extracellular matrix protein Reelin is involved in the development of GCD, since the Reelin-deficient *reeler* mouse (D’Arcangelo et al., 1995; Hirotsune et al., 1995) shows a disorganized GCL, reminiscent of GCD (Frotscher et al., 2003). Moreover, GCD formation has been shown to be accompanied by a loss of Reelin-producing neurons in the hippocampus of MTLE patients (Haas et al., 2002) and in rodent epilepsy models (Heinrich et al., 2006; Gong et al., 2007; Antonucci et al., 2008; Duveau et al., 2011). A local reduction of GCD has been achieved by infusion of recombinant Reelin into the rodent hippocampus during epileptogenesis, pointing to a causal role of Reelin in maintaining lamination in the adult brain (Müller et al., 2009).

Reelin is a key regulator of neuronal positioning during brain development, but Reelin is also important for synaptic function and memory formation in the adult brain (Herz and Chen, 2006; Lane-Donovan et al., 2015). Reelin is synthesized and secreted by Cajal-Retzius (CR) cells and interneurons into the extracellular matrix (Alcántara et al., 1998; Pesold et al., 1998), where full-length Reelin is proteolytically cleaved into smaller isoforms, an important prerequisite for activation of target cells (Jossin et al., 2004, 2007). The full-length Reelin molecule consists of an N-terminal F-spondin-like sequence, followed by eight Reelin repeats. Cleavage of Reelin can occur at two sites: N-terminal between the second and the third repeat, and C-terminal between the sixth and seventh repeat, generating five possible isoforms depending on the protease in action (Lambert de Rouvroit et al., 1999; Jossin et al., 2004). Specifically, the central region of Reelin (R3–6) has been described to be very important for receptor binding (D’Arcangelo et al., 1999; Jossin et al., 2004; Lee and D’Arcangelo, 2016).

Reelin signaling occurs after binding to lipoprotein receptors, very-low-density lipoprotein receptor (VLDLR) and apolipoprotein E receptor 2 (ApoER2), tyrosine phosphorylation of the intracellular adaptor protein Disabled-1 (Dab1) by Src family kinases, and the subsequent activation of downstream effectors, which target the actin and microtubule cytoskeleton (Tissir and Goffinet, 2003; Stolt and Bock, 2006; Jossin and Goffinet, 2007; Leemhuis and Bock, 2011). Recently, binding of Reelin to Ephrins has been reported, although the physiological functions of these interactions remain poorly understood (Sentürk et al., 2011; Bouché et al., 2013).

Granule cell dispersion can be induced experimentally in adult mice by unilateral injection of kainate (KA), an agonist of the excitatory neurotransmitter glutamate (Bouilleret et al., 1999; Heinrich et al., 2006; Häussler et al., 2012). In this animal model, AHS including neuronal cell loss and GCD develops within 3 weeks after KA injection in spite of the loss of dentate neurogenesis (Kralic et al., 2005; Heinrich et al., 2006; Nitta et al., 2008). In addition, spontaneous, focal epileptic seizures develop similar to human MTLE (Riban et al., 2002; Häussler et al., 2012). GCD can also be induced *in vitro* in organotypic hippocampal slice cultures (OHSCs) by KA application (Tinnes et al., 2011, 2013). In this *in vitro* model, GCD has been shown recently to occur via somal translocation of differentiated granule cells (Murphy and Danzer, 2011; Chai et al., 2014), but so far the molecular mechanism has remained unclear.

In the present study, we show in the intrahippocampal KA mouse model that GCD formation and loss of reelin mRNA-producing neurons are spatially correlated and that this loss mainly affects the hilus. In addition, we present evidence in OHSC that, like *in vivo*, KA treatment causes a complete loss of Reelin-producing hilar neurons. Moreover, we show in real time by life cell microscopy that differentiated enhanced green fluorescent protein (eGFP)-positive granule cells actively migrate toward the Reelin-free hilar region and that this migration process can be prevented by application of the central R3–6 Reelin fragment.

## Materials and Methods

### Animals

Experiments were performed with C57BL/6 and Thy1-eGFP mice (M-line, C57BL/6 background). All animal procedures were carried out in accordance with the guidelines of the European Community’s Council Directive of 22 September 2010 (2010/63/EU) and were approved by the regional council (Regierungspräsidium Freiburg).

### Intrahippocampal Kainate Injection

Adult male (7–12 weeks of age) C57BL/6 mice were used for unilateral intrahippocampal KA injections as described previously (Heinrich et al., 2006; Häussler et al., 2012). In brief, anesthetized mice (ketamine hydrochloride 100 mg/kg, xylazine 5 mg/kg, atropine 0.1 mg/kg body weight, i.p.) were stereotaxically injected with 50 nl (1 nmol) of a 20 mM KA solution (Tocris) in 0.9% sterile saline into the right dorsal hippocampus [coordinates relative to bregma: anterio-posterior (AP) = -2.0 mm, medio-lateral (ML) = -1.4 mm, dorso-ventral (DV) = -1.8 mm]. Controls were injected with 0.9% saline. After recovery from anesthesia, mice were kept under observation for several hours. Behavioral *status epilepticus* (SE) was verified, characterized by mild convulsive movements, chewing, rotations or immobility, as previously described (Riban et al., 2002; Heinrich et al., 2006). Only mice that had experienced SE after KA injection were kept for further experiments.

### *In situ* Hybridization

Localization of reelin mRNA was performed by *in situ* hybridization (ISH) with digoxigenin (DIG)-labeled cRNA probes as described earlier (Haas et al., 2002; Heinrich et al., 2006). Three weeks after KA injection, mice were deeply anesthetized (see above), transcardially perfused for 10 min with paraformaldehyde (PFA) in 0.1 M phosphate buffer (PB), pH 7.4, followed by post-fixation of isolated brains for 4 h at 4°C in PFA, cryoprotection (20% sucrose in PB overnight at 4°C) and sectioning (50 μm; coronal plane) on a cryostat. Slices were collected in culture dishes containing 2x SSC (1x SSC = 0.15 M NaCl and 0.015 M sodium citrate, pH 7.0), followed by incubation with a 1:1 mixture of 2x SSC and hybridization buffer (50% formamide, 4x SSC, 50 mM NaH_2_PO_4_, 250 μg/ml heat-denaturated salmon sperm DNA, 100 μg/ml tRNA, 5% dextransulfate, 1% Denhardt’s solution) for 15 min. Prehybridization in hybridization buffer for 50 min at 45°C was followed by hybridization in the same buffer supplemented with DIG-labeled antisense reelin cRNA probe (100 ng/ml) at 45°C. Brain sections were washed twice in 2x SSC, followed by stringent washing at 55°C with 2x SSC and 50% formamide, 0.1x SSC and 50% formamide, and 0.1x SSC. Immunological detection of the hybrids was performed with an anti-DIG antibody conjugated with alkaline phosphatase and nitroblue tetrazolium and 5-bromo-4-chloro-3-indolyl phosphate as substrates. Tissue sections were mounted on slides, air dried, and embedded in Kaiser’s gelatine (Roche).

### Quantification of Reelin mRNA-Expressing Neurons and Correlation with GCL Width

Reelin mRNA-expressing neurons were quantified in the whole hippocampus of control animals (NaCl-injected) and after KA injection. Cells were counted at three positions along the septohippocampal axis (septal, intermediate, and temporal). Cell numbers were determined in two regions of interest (ROI) per section: ROI1 included the *strata moleculare, lacunosum moleculare* and *radiatum*, whereas ROI2 comprised the hilus (see Supplementary Figure S1). All reelin mRNA-expressing neurons were counted in each ROI using the *ImageJ* analysis software (NIH, public domain). Cell densities were determined in three sections per position by relating cell numbers to the area of the respective ROI. These data were imported to *GraphPad Prism 5* software for statistical analysis.

Next, the mean GCL thickness was determined in the same tissue sections used for the quantification of reelin mRNA-producing neurons. Both GCL blades were subdivided into portions with constant thickness (see Supplementary Figure S2). Their width was measured with *ImageJ*. The respective values were multiplied with the length of the respective GCL portion. The multiplication-products from all CGL parts were summed up and divided by the total GCL length: (Length_1_ × Width_1_ + Length_2_ × Width_2_… + Length_n_ × Width_n_)/Total Length. Finally, the mean GCL thickness was correlated with the corresponding density of reelin mRNA-positive neurons in the hilus using *GraphPad Prism 5* software. We fitted the curve of a non-linear regression analysis (exponential growth equation) into the data points, since we found this equation to be the most suitable approximation to the real data.

### Organotypic Hippocampal Slice Cultures

Eight-days-old (P8) male and female Thy1-eGFP mouse pups were used for OHSC preparation as described previously (Chai et al., 2014). In brief, brains were removed from the skull following decapitation under isoflurane anesthesia. The hippocampi were dissected and sliced (400 μm) perpendicular to the longitudinal axis of the hippocampus using a *McIlwain* tissue chopper. Only slices from the mid region of each hippocampus were used. The slices were placed onto culture inserts (Millipore) and transferred to 6-well plates with 1 ml/well of nutrition medium containing 50% minimal essential medium (MEM), 25% basal medium Eagle (BME), 25% heat-inactivated horse serum (Invitrogen) supplemented with 0.65% glucose and 2 mM glutamate (pH 7.2). OHSC were incubated as static cultures (Stoppini et al., 1991) in 5% CO_2_ at 37°C for 7 days *in vitro* (DIV) before experiments started; the medium was changed every second day.

### Production and Purification of the Recombinant Reelin Fragment R3–6

Human embryonic kidney 293 (HEK 293) cells were grown to subconfluency and were transiently transfected with an expression vector containing the myc-tagged R3–6 Reelin fragment (central fragment; Jossin et al., 2004; Bouché et al., 2013). One day after transfection, the fetal calf serum (FCS)-containing cell culture medium was replaced by serum-free Dulbecco’s modified Eagle’s medium (DMEM; Invitrogen) supplemented with 1.0 g/l glucose. After 2 DIV, the cell culture medium containing the R3–6 Reelin fragment was harvested and concentrated by centrifugation using 100 kDa cutoff centrifugal filters (Merck Millipore), sterile filtered, and stored at -20°C until further use (Leemhuis et al., 2010).

Purity and size of the recombinant R3–6 Reelin fragment was confirmed by Western blot analysis. To this end, recombinant full length Reelin and R3–6 Reelin were size-fractionated by a 3–8% Tris-acetate sodium dodecyl sulfate-polyacrylamide gel electrophoresis (SDS-PAGE; Invitrogen) and blotted onto a polyvinylidene fluoride (PVDF) membrane (Roche). For immunodetection, the PVDF membrane was blocked with I-Block buffer (Tropix) and incubated with the monoclonal Reelin antibody R4B (2:1), produced in our laboratory as described by Jossin et al. (2007). It was followed by the incubation with the appropriate alkaline-phosphatase-conjugated secondary antibody (1:10,000; Tropix) and CDP Star (Tropix) was used as substrate for chemiluminescent detection by the *Chemismart* System (Peqlab Biotechnologies; see Supplementary Figure S3).

### Concentration Assessment of the Recombinant R3–6 Reelin Fragment

The concentration of the recombinant R3–6 Reelin fragment was assessed by a dot blot assay followed by immunofluorescence detection. In brief, recombinant mouse Reelin protein with a known concentration (100 μg/mL, R&D Systems) and purified recombinant R3–6 Reelin were serially diluted and spotted onto nitrocellulose membrane (Santa Cruz Biotechnology). For immunodetection, the membrane was probed with the same antibody as for the Western blot, followed by incubation with the respective alkaline-phosphatase-conjugated secondary antibody (1:10,000; Tropix). CDP Star (Tropix) was used as substrate for chemiluminescent detection by the *Chemismart* System. Densitometric evaluation of the dot blot signals was performed by optical density (OD) measurement using *ImageJ* software. The concentration was calculated based on the comparison of OD values obtained for the recombinant mouse Reelin standard curve and the recombinant R3–6 Reelin fragment.

### Immunohistochemistry

Tissue sections (50 μm) from perfused KA-injected mouse brains (see above) or whole OHSC from Thy1-eGFP mice were immunolabeled using a free-floating protocol (Heinrich et al., 2006; Tinnes et al., 2011). OHSC (13 DIV) were fixed with 4% PFA in PB (4 h, RT). After pre-incubation (0.25% Triton X-100, 10% normal serum in PB, 2 h), tissue sections or slices were incubated for 24 h (RT) with mouse monoclonal anti-Reelin antibody (G-10, 1:1000; Chemicon) or rabbit polyclonal anti-Prox1 (1:1000, Abcam). Antibody binding was visualized by incubation with an appropriate Cy3-conjugated secondary antibody (1:400, Jackson ImmunoResearch Laboratories) in the dark (6 h, RT). Tissue sections were counterstained with DAPI. Sections and whole slices were coverslipped with anti-fading mounting medium (IMMU-Mount, Thermo Fisher Scientific) and analyzed using an epifluorescence (Axio Imager 2, Carl Zeiss) or confocal microscope (Olympus FluoView FV10i).

### Quantification of Reelin-Immunofluorescence in OHSC

Reelin-expressing neurons were quantified in whole slices under control conditions and after KA (10 μM) application for 45 min, followed by incubation in fresh medium for 8 h. To quantify Reelin fluorescence intensity, confocal z-stacks were acquired from whole OHSC with an Olympus FV10i confocal laser scanning microscope (Olympus) using a 10x objective at high resolution (1024 × 1024 pixel; 8× frame-average) with constant exposure time and z-stack settings. Stacks were converted to grayscale and the signal intensity of Reelin immunolabeling along the hippocampal fissure (HF) and in the hilus was quantified as integrated density using the *ImageJ* analysis software. Values were corrected by background subtraction: integrated density – (measured area × mean background signal). The mean background was calculated for each experiment and for both areas of the HF and hilus. Background signals were measured in areas without Reelin signal.

### Live Cell Imaging

Organotypic hippocampal slice cultures were prepared from Thy1-eGFP mouse pups (P8) and cultivated for 7–18 DIV. Immediately before live cell imaging, OHSC were exposed to KA (3 or 10 μM) for 45 min, followed by addition of fresh medium or fresh medium including recombinant R3–6 Reelin fragment (1 nM) followed by live cell imaging for 8 h. For imaging, OHSC were placed, on the stage of a Leica SP2 confocal microscope, and enclosed in an aerated chamber at 37°C and with humidified atmosphere containing 5% CO_2_. eGFP-positive granule cells were imaged along the Z-axis with a spacing of 6 μm, using a 10x objective at 2x optical zoom, at 45 min intervals over a period of 8 h. To monitor the positions of individual granule cells, confocal image stacks taken at the different time points were imported into the image analysis software *Fiji*, which is based on *ImageJ* (NIH, public domain). Using the *MtrackJ*© plugin (Erik Meijering), six individual granule cells/OHSC were randomly selected. Their positions were marked at all 10 time points over a period of 8 h to allow for the calculation of their migration distances.

To investigate preferred directions of migration, we identified individual granule cells in the superficial and deep GCL (three cells per sublayer/OHSC) after exposure to KA or control conditions. Cell motility was quantified by calculating the total length of the traveled path over 8 h and the effective distance of the migration as the length of the resulting vector between start and end point of the cell. To differentiate the direction of migration, we marked the border between the hilus (H) and GCL and calculated the distance of the start and end point of the cells to this hilar-GCL border. Cells that reduced their distance to the border over time [H_distance_(*t* = 0 min) – H_distance_(*t* = 450 min)] were considered to migrate toward the hilus, whereas cells showing an increased distance in relation to the hilar-GCL border moved toward the molecular layer (ML). We used custom Matlab^®^ software to perform this data analysis (Matlab^®^ 2014a, The-Mathworks).

### Statistical Analysis

All values are expressed as mean ± standard error of the mean (SEM). All statistical analyses were performed with *GraphPad Prism 5* software. Differences between groups were tested for statistical significance (Student’s *t*-test or one-way ANOVA with Tukey’s multiple comparison test). Significance levels were set to ^∗^*P* < 0.05, ^∗∗^*P* < 0.01, and ^∗∗∗^*P* < 0.001.

## Results

### Quantification of Reelin mRNA-Expressing Neurons Along the Septotemporal Axis After Intrahippocampal KA Injection

In previous studies, a loss of reelin-producing neurons has been shown in the septal hippocampus after KA injection on the mRNA and protein level (Heinrich et al., 2006; Antonucci et al., 2008; Duveau et al., 2011). Yet, these reports focused only on the area close to the KA injection site. There is, however, a septotemporal gradient of GCD and cell loss in the intrahippocampal KA mouse model (Häussler et al., 2012; Marx et al., 2013). Therefore, we aimed at precisely determining the spatial distribution of reelin-expressing neurons along the septotemporal axis of the hippocampus after KA injection by ISH and immunohistochemistry. To this end, reelin mRNA-positive neurons were counted in tissue sections at three positions (septal, intermediate, and temporal) along the hippocampal septotemporal axis at 21 days after KA injection, a time point when GCD has fully developed (Heinrich et al., 2006) and we correlated the GCL width with the number of reelin mRNA-positive hilar neurons. NaCl-injected mice were used as controls (see Materials and Methods, Supplementary Figure S1).

**FIGURE 1:**
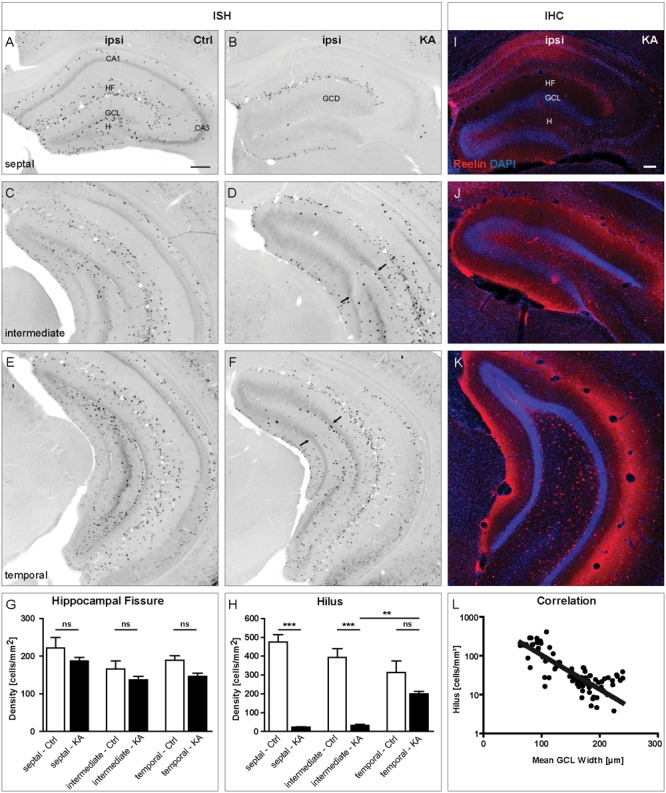
**Loss of reelin mRNA-expressing neurons coincides with GCD formation after unilateral intrahippocampal KA injection in adult mice.** ISH for reelin mRNA was performed on tissue sections along the septotemporal axis at 21 days after KA injection. **(A–F)** Representative images showing the distribution of reelin mRNA-positive neurons in sections of the septal **(A,B)**, intermediate **(C,D)** and temporal **(E,F)** portions of the hippocampus of control animals **(A,C,E)** and ipsilateral to the KA injection **(B,D,F)**. **(A,C,E)** In controls, many reelin mRNA-expressing neurons are present in the *stratum oriens, stratum lacunosum moleculare*, along the HF and in the hilus at all three positions along the septotemporal axis of the hippocampus (septal, intermediate, and temporal). **(B)** At 21 days after intrahippocampal KA injection there is a drastic loss of reelin mRNA-expressing neurons all over the ipsilateral septal hippocampus, where GCD is present. Only at the HF, they are preserved. **(D,F)** A loss of reelin mRNA-positive neurons can also be observed in intermediate and temporal sections, however, being confined to the portion with GCD. Arrows indicate the transition from GCD to normal GCL, where reelin mRNA-positive neurons reappear. **(G,H)** Quantification of the number of reelin mRNA-expressing neurons in the HF **(G)** and the hilus **(H)** in controls (*n* = 3) and after KA injection (*n* = 5) at three different positions along the septotemporal axis of the hippocampus (septal, intermediate, and temporal). Note the significant loss of reelin mRNA-expressing neurons in the hilus at septal and intermediate positions. One-way ANOVA followed by Tukey’s Multiple Comparison Test (^∗∗^*P* < 0.01; ^∗∗∗^*P* < 0.001). Representative images of tissue sections immunolabeled for Reelin (red) counterstained with DAPI (blue) of the septal **(I)**, intermediate **(J)**, and temporal **(K)** portions of the hippocampus ipsilateral to the KA injection. Like on the mRNA level, Reelin-expressing neurons are gone in the septal and intermediate hilus but are preserved at the HF. They reappear in the temporal hippocampus, mirroring the distribution of reelin mRNA expression. **(L)** Graph showing the correlation of the mean GCL width with the number of reelin mRNA-positive hilar neurons (*r*^2^ = 0.6112). CA1, *cornu ammonis* 1; CA3, *cornu ammonis* 3; HF, hippocampal fissure; GCL, granule cell layer; H, hilus; ML, molecular layer. Scale bars: 200 μm.

In tissue sections of control animals, many reelin mRNA-expressing neurons were observed in *stratum oriens, stratum lacunosum moleculare* along the HF and in the hilus at all positions along the septotemporal axis of the hippocampus (**Figures 1A,C,E**). After KA injection, a drastic loss of reelin mRNA- and Reelin-expressing neurons was evident all over the ipsilateral septal hippocampus, only at the HF, reelin mRNA- and Reelin-positive neurons were preserved (**Figures 1B,I**). Interestingly, this loss was confined to the septal portion of the hippocampus, where GCD was present (**Figures 1B,D,F,I–K**). Cell counting revealed that the density of reelin mRNA-expressing neurons was decreased ipsilaterally at the HF at all three positions when compared to controls (septal; control: 228.5 ± 31.26, KA: 194.0 ± 12.6; intermediate; control: 165 ± 24.45, KA: 132 ± 7.1; temporal; control: 188 ± 9.9, KA: 147.0 ± 8.6 cells/mm^2^), but this decrease did not reach significance (**Figures 1G**). In the hilus, however, an almost complete loss of reelin mRNA-synthetizing neurons was detectable in sections of the septal and intermediate KA-injected hippocampus when compared to controls (septal; control: 469.3 ± 69.8, KA: 22.92 ± 3.2; intermediate; control: 413.2 ± 56.1, KA: 27.56 ± 5.9 cells/mm^2^), whereas at the temporal position the loss was not significant in comparison to controls (temporal; control: 335.9 ± 71.9, KA: 209.6 ± 15.56 cells/mm^2^; **Figure 1H**). Next, we determined the GCL width ipsilaterally in the same tissue sections and related it to the number of reelin mRNA-positive hilar neurons. We found an inverse correlation of GCL width and number of reelin mRNA-expressing neurons in the hilus (**Figure 1L**).

In summary, our detailed quantification along the septotemporal axis revealed that the loss of reelin mRNA-positive neurons was strongest in the hilus in regions exhibiting GCD and this loss precisely correlates with the occurrence of GCD.

### Distribution of Reelin-Expressing Neurons in KA-Treated OHSC

**FIGURE 2:**
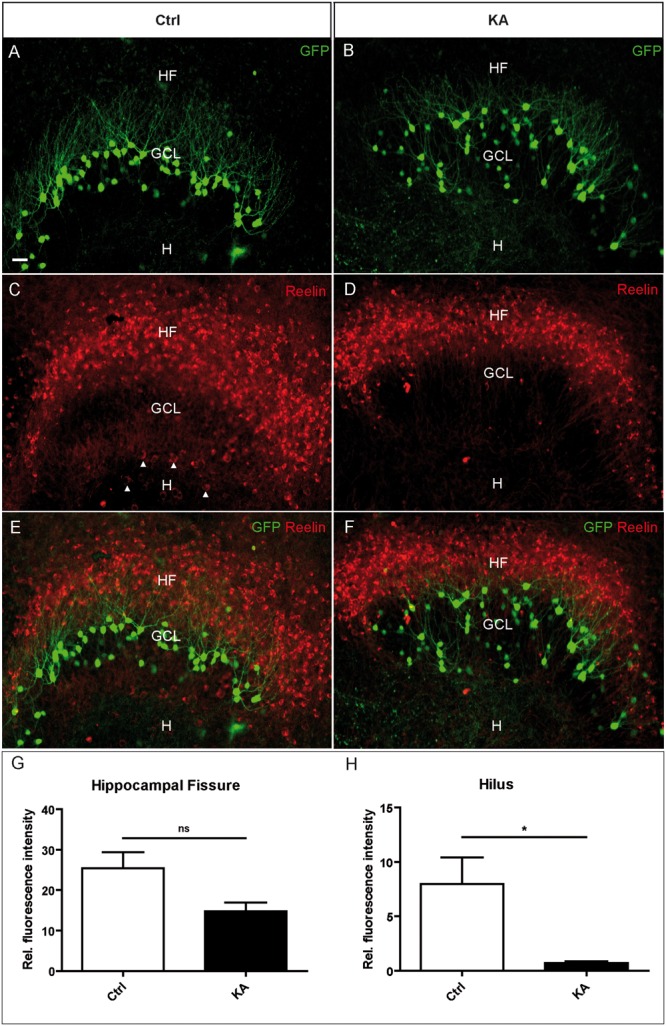
**Loss of Reelin-positive neurons in the hilus after KA treatment in OHSC.** Representative confocal images of whole OHSC from Thy1-eGFP (enhanced green fluorescent protein; green) mice immunolabeled for Reelin (red). **(A,C,E)** Control. **(B,D,F)** OHSC, 8 h after KA application for 45 min. In the control, eGFP-positive granule cells are arranged in a compact layer **(A)** but appear dispersed after KA treatment **(B)**. **(C,D)** Immunolabeling for Reelin. Many strongly immunostained Reelin-expressing neurons are visible at the HF, and large Reelin-immunopositive neurons are located in the hilus (arrow heads, **C**). Note the loss of Reelin-positive hilar interneurons after KA treatment **(D)**. **(E,F)** Overlay of eGFP (green) and Reelin (red) signals. **(G,H)** Densitometric quantification of the Reelin signal at the HF **(G)** and in the hilus **(H)** in controls (*n* = 4) and after KA treatment (*n* = 4). Unpaired student’s *t*-test (^∗^*P* < 0.05, ns with *P* = 0.057). GCL, granule cell layer; H, hilus; HF, hippocampal fissure. Scale bar: 10 μm.

Next, we used OHSC from transgenic Thy1-eGFP mice to investigate the effect of the KA treatment on Reelin-producing neurons *in vitro*. KA (10 μm) was applied to OHSC for 45 min, followed by incubation in fresh medium for 8 h. Slices were fixed with PFA and whole OHSC were immunolabeled for Reelin. In control OHSC, eGFP-positive granule cells were densely arranged in a compact layer with dendrites extending into the ML; Reelin-positive neurons were located at the HF and in the hilus (**Figures 2A,C,E**). In KA-treated OHSC, eGFP-positive granule cells appeared dispersed; there were still many Reelin-immunolabeled neurons at the HF, but almost all had disappeared from the hilus (**Figures 2B,D,F**). Densitometric evaluation revealed a slight reduction of Reelin immunofluorescence at the HF, but an almost complete, significant loss of the Reelin signal in the hilus (control: HF 25.46 ± 3:9; H 7.99 ± 2.4; KA: HF 14.83 ± 2.1; H 0.7 ± 0.18; **Figures 2G,H**). These results show that, like *in vivo*, Reelin-expressing neurons were lost in the hilus, whereas Reelin-synthetizing neurons at the HF survived.

### Life Cell Imaging of eGFP-Positive Granule Cells in OHSC After Treatment with Increasing Doses of KA

**FIGURE 3:**
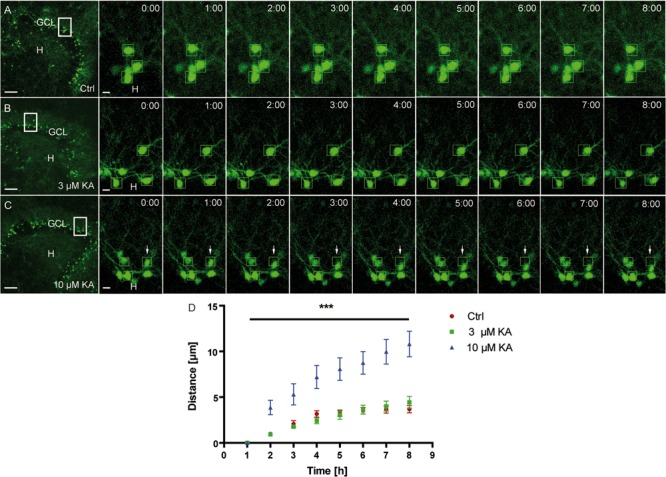
**Motility of granule cells depends on the KA concentration.** Life cell imaging of individual eGFP-labeled granule cells was performed over a period of 8 h, and migration distances of randomly selected cells were assessed as described in Section “Materials and Methods.” Representative confocal micrographs of nine imaging time points are shown for each condition. Left panel: overview; white frame indicates the area shown at high magnification on the right. Tracked cells, are marked by green frames. **(A)** Control (*n* = 4; 24 cells). Cells remain in place. **(B)** 3 μM KA (*n* = 4; 24 cells). Tracked cells behave similar to controls. **(C)** 10 μM KA (*n* = 3, 18 cells). Granule cells show increased motility (white arrows). **(D)** Statistical analysis of the motility of adult granule cells of control and KA-treated OHSC (3 μM KA, 10 μM). The migration behavior increases in a KA dose-dependent manner over the entire 8 h period. One-way ANOVA followed by Tukey’s test with (^∗∗∗^*P* < 0.001). GCL, granule cell layer; H, hilus. Scale bars: 80 μm for overview; 5 μm for high magnifications.

As a prerequisite for later life cell imaging experiments, we first determined the minimal KA concentration needed to trigger migration. To this end, OHSC from Thy1-eGFP mice were exposed to 3 or 10 μM KA for 45 min, followed by incubation in fresh medium and life cell imaging for 8 h (**Figures 3A–C**). Afterward, the migration distances of individual eGFP-labeled granule cells were determined (**Figure 3D**). We found that 3 μM KA (4.43 ± 0.63 μm) increased the motility of eGFP-labeled granule cells only slightly above control values (3.68 ± 0.4 μm) during the observation period, whereas 10 μM KA (10.8 ± 1.4 μm) triggered the movement significantly. Hence, we used this KA concentration for all subsequent imaging experiments.

### Migration Behavior of eGFP-Positive Granule Cells in OHSC After KA Treatment

**FIGURE 4:**
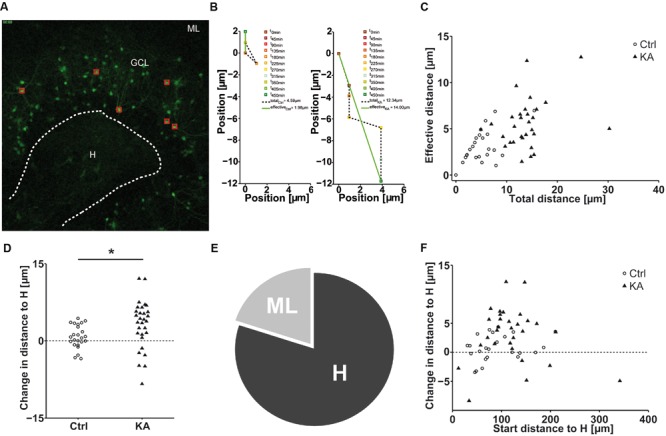
**EGFP-positive granule cells migrate preferentially to the hilus.** Life cell imaging of individual eGFP-labeled granule cells in the superficial and deep granule cell layer after exposure to KA and in controls over a period of 8 h. **(A)** Representative photomicrograph of a KA-treated OHSC from a Thy-1 eGFP mouse (*t* = 0 min). Red squares indicate the initial position of monitored cells. White dashed line marks the border between the hilus and the GCL, taken as reference to calculate the relative migration distances of individual eGFP-labeled cells. **(B)** Representative graphs showing the motility of one tracked neuron in a control (left graph) or a KA-treated OHSC (right graph). Cell motility was assessed by calculating both, the total length of the traveled path over 8 h (black dashed line = total distance) and the effective distance of the migration as the length of the resulting vector (green solid line) between start (at *x* = 0; *t* = 0 min) and end point (*t* = 450 min). **(C)** Diagram showing the relation between total and effective migration distance of all tracked granule cells. In contrast to controls, all cells change their position after KA treatment. **(D)** Migration distances of all tracked individual granule cells with respect to the border between hilus and GCL in control (*n* = 4, 24 cells) and KA-treated OHSC (*n* = 5, 30 cells). To differentiate the migration direction, we marked the border between the hilus and GCL (**A**, white dashed line) and calculated the distance of the start and end point of the cells to this border. Cells that reduce their distance to the border over time [H_distance_ (*t* = 0 min) – H_distance_ (*t* = 450 min), positive values] are considered migrating toward the hilus. Cells showing an increase in distance (negative values) moved toward the ML. Cells in KA-treated OHSC (black triangles) travel longer distances in both directions, toward ML and hilus, than controls (black circles). Two-sample Student’s *t*-test (^∗^*P* < 0.05). Cells which migrate toward the hilus travel longer distances than those heading toward the ML. The majority of cells progresses toward the hilus. **(E)** Relative distribution of cells traveling toward the hilus (80%) or molecular layer (20%) in KA-treated OHSC. **(F)** Diagram showing the relation between initial distance to the hilus-GCL border and change in distance to the border of all tracked granule cells. In contrast to controls, all cells change their position after KA treatment and travel longer distances regardless of their position in the deep or superficial granule cell layer. ML, molecular layer; GCL, granule cell layer; H, hilus.

Next, we aimed at investigating whether eGFP-labeled granule cells followed a specific migration pattern triggered by KA treatment. As described above, we performed life cell imaging after KA (10 μM) treatment and compared it to untreated controls. We randomly marked individual granule cells in the superficial and deep GCL (three cells per sublayer/OHSC) and tracked their position with respect to the border between the hilus and GCL (**Figure 4A**) over a period of 8 h. We quantified differences in motility by calculating (1) the total length of the traveled path over 8 h and (2) the effective distance of migration as the length of the vector between start (*t* = 0 min) and end point (*t* = 450 min; **Figure 4B**).

This analysis revealed that after KA treatment granule cells showed a significantly higher motility than under control condi tions with an increased total path length (Ctrl = 5.10 ± 0.65 μm; KA = 13.86 ± 0.84 μm; *P* < 1.73 × 10^-10^) and an increased effective distance (Ctrl = 3.10 ± 0.36 μm; KA = 5.59 ± 0.49 μm; *P* < 2.6 × 10^-4^). Furthermore, we found that the total path length increased correspondingly with the length of the effective path (**Figure 4C**). In addition, we observed that the majority of granule cells from KA-treated slices (80%) migrated toward the hilus (**Figures 4D,E**), regardless of their initial position within the GCL (**Figure 4F**). Moreover, cells migrating toward the hilus traveled longer distances than those heading to the ML (changed distance to hilus, Ctrl = 0.76 ± 0.46 μm; KA = 3.17 ± 0.85 μm; *P* < 0.02; **Figure 4C**).

### Influence of the Recombinant R3–6 Central Reelin Fragment on KA-Induced Motility of eGFP-Labeled Granule Cells

So far, our results showed that in KA-treated OHSC Reelin-expressing neurons are mainly lost in the hilus and that the majority of eGFP-positive granule cells migrate toward the hilus. Together, these findings point to a stop signal function of Reelin which might be lost in the presence of KA. Thus, we next investigated whether the R3–6 central Reelin fragment, known to be important for activation of the Reelin signaling cascade (Jossin et al., 2004), would be capable of preventing the observed KA-triggered movement of granule cells. For this purpose, OHSC were treated for 45 min with KA, followed by washout, addition of the recombinant R3–6 Reelin fragment to the medium and subsequent live cell imaging for 8 h.

**FIGURE 5:**
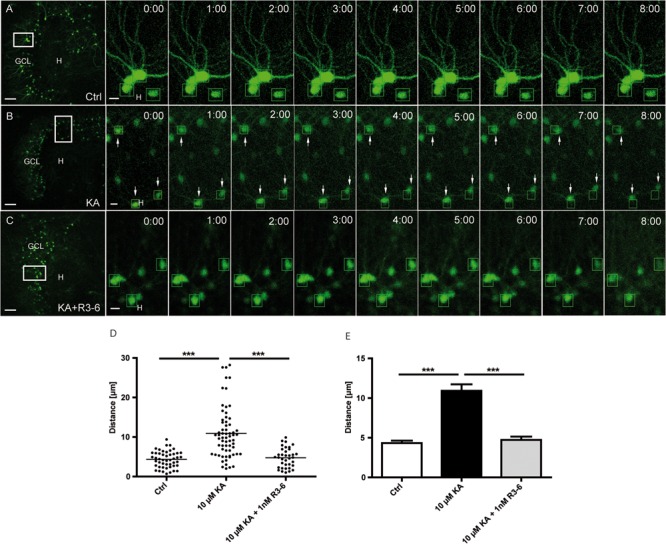
**Kainate-induced migration of differentiated granule cells is prevented by the central R3–6 Reelin fragment.** Live cell imaging of individual eGFP-positive granule cells was performed over a period of 8 h, and migration distances of randomly selected cells were assessed as described in Section “Materials and Methods.” Representative confocal micrographs of nine imaging time points are shown for each condition. Left panel: overview; white frame indicates the area shown at high magnification on the right. Tracked cells are marked by green frames. **(A)** Untreated control (*n* = 8; 48 cells). Cells do not change position. **(B)** Treatment of OHSC with 10 μM KA for 45 min (*n* = 10; 60 cells). Granule cells (GCs) with high motility are indicated by arrows. **(C)** Treatment of OHSC with 10 μM KA for 45 min, followed by incubation with fresh medium and subsequent application of recombinant R3–6 Reelin fragment (1 nM; *n* = 6; 36 cells). Like controls, cells do not change position. **(D,E)** Quantitative evaluation of migration distances of individual GCs. KA-treated GCs migrate, but not in the presence of the recombinant R3–6 Reelin fragment. One-way ANOVA, followed by Tukey’s Multiple Comparison Test (^∗∗∗^*P* < 0.001; Ctrl vs. 10 μM KA + 1 nM R3–6 shows no significance). GCL, granule cell layer; H, hilus. Scale bars: 80 μm for overview; 5 μm for high magnifications.

As shown before, KA treatment alone caused an increased motility (10.4 ± 1.4 μm) of granule cells when compared to the controls (3.9 ± 0.4 μm; **Figures 5A,B**). In contrast, the presence of the R3–6 Reelin fragment significantly prevented the movement of granule cells (4.2 ± 0.5 μm) when compared with KA-treated OHSC (**Figures 5C–E**). As a control, we showed that the application of KA and the R3–6 Reelin fragment did not prevent the KA-mediated loss of Reelin-positive hilar neurons (see Supplementary Figure S4). These results indicate that the central Reelin fragment was able to stop the KA-mediated migration of eGFP-positive granule cells and support the role for Reelin as a positional signal for granule cells in the adult hippocampus.

## Discussion

In this study, we show that reelin-producing neurons are mainly lost in the hilus after intrahippocampal KA injection in mice and that this loss correlates precisely with the occurrence of GCD. Also in OHSC, KA treatment causes a rapid and complete loss of hilar, Reelin-producing neurons. Using life cell microscopy, we provide evidence that differentiated eGFP-positive granule cells actively migrate toward the Reelin-free hilar region, and that this migration process can be prevented by application of the recombinant R3–6 Reelin fragment. Thus, the development of the GCD seems to depend on the loss of functionally active Reelin.

### Loss of Reelin mRNA-Expressing Neurons Correlates Spatially with the Occurrence of GCD in the Epileptic Mouse Hippocampus

In the hippocampus reelin mRNA and protein is mainly expressed by GABAergic interneurons located primarily in *stratum oriens* and *radiatum* of *cornu ammonis* (CA) 1 and CA3 and in the hilus of the dentate gyrus but also by CR cells located along the HF (Alcántara et al., 1998; Pesold et al., 1998; Ramos-Moreno et al., 2006). In the present study, we show by detailed quantification of reelin mRNA expression along the septotemporal axis of the epileptic mouse hippocampus that reelin mRNA-synthetizing CR cells at the HF survive, whereas in the hilus reelin mRNA-expressing interneurons are lost. In fact, this loss is confined to the septal hippocampus and precisely matches spatially with the occurrence of GCD, as demonstrated by correlating GCL width and numbers of reelin-synthesizing neurons. These results confirm and extend previous studies in the same epilepsy model, showing that Reelin-producing CR cells are preserved at the HF but lost in the hilus (Heinrich et al., 2006; Antonucci et al., 2008; Duveau et al., 2011). However, all these reports focused only on the area close to the KA injection site. Here, we demonstrate that the loss of Reelin synthesis precisely mirrors the septotemporal gradient of GCD described for this epilepsy model (Häussler et al., 2012) and highlight a link between Reelin loss and GCD formation.

### KA Treatment of OHSC Causes a Rapid Loss of Reelin-Producing Neurons in the Hilus

Organotypic hippocampal slice cultures have been shown to be a suitable model to study neuronal changes induced by epileptiform activity. Challenged by treatment with the glutamate receptor agonist KA, OHSC develop histopathological features similar to AHS such as cell death, mossy fiber sprouting, GCD, and epileptic activity (Routbort et al., 1999; Tinnes et al., 2011; Chai et al., 2014). Here we used OHSC obtained from Thy1-eGFP mice, known to express eGFP primarily in a subset of differentiated granule cells as shown previously (Feng et al., 2000; Chai et al., 2014) and by double labeling with Prox1, a marker for differentiated granule cells (Supplementary Figure S5). When OHSC were exposed to KA for 45 min, a rapid (within 8 h) and selective loss of Reelin-positive interneurons was observed in the hilus, whereas Reelin-positive CR cells persisted. This observation confirms the high vulnerability of Reelin-producing interneurons to KA-mediated excitotoxicity seen *in vivo* and reported in OHSC previously (Tinnes et al., 2011; Chai et al., 2014), resulting in a Reelin loss confined to the hilar region. The observed difference in survival rate between interneurons and CR cells is most likely due to differential expression of glutamate receptors, since only hilar interneurons, but not CR cells, up-regulate c-Fos after KA treatment (Tinnes et al., 2011).

### eGFP-Positive Granule Cells Preferentially Migrate toward the Reelin-Poor Hilus

In the adult dentate gyrus, granule cells form a densely packed layer. Under epileptic conditions, the lamination can dissolve and result in GCD as observed, in MTLE patients (Houser, 1990; Haas et al., 2002) and in our MTLE mouse model (Bouilleret et al., 1999; Heinrich et al., 2006; Häussler et al., 2012). GCD formation is a process affecting differentiating granule cells, since GCD develops in the absence of neurogenesis as shown previously after intrahippocampal KA injection (Kralic et al., 2005; Heinrich et al., 2006; Nitta et al., 2008). Recent *in vitro* studies reported that this migration process is based on somal translocation (Murphy and Danzer, 2011; Chai et al., 2014). Somatic translocation is a principal migratory mechanism of neurons during brain development and occurs when the nucleus and perisomatic cytoplasm are displaced into a leading process (Rakic, 1972; Métin et al., 2008). Here, we show by life cell microscopy that eGFP-labeled, differentiated granule cells become motile in response to KA challenge. By tracking the path of individual neurons during the whole observation period, we found that the majority (80%) moved in the direction of the hilus, whereas only 20% traveled to the opposite direction toward the ML. The migration pattern appeared rather complex. The neurons did not move straight into one direction but traveled erroneously in different directions before they reached a position closer to the hilar (or ML) area. It is tempting to speculate that most of the granule cells moved specifically to the direction of the Reelin-free hilar area. We cannot exclude, however, that a functional inactivation of Reelin by impaired proteolytic processing, known to occur under epileptic conditions (Tinnes et al., 2011, 2013), might play a role in the movement of neurons toward the ML.

### KA-Induced Motility of Adult Granule Cells is Prevented by Application of the Central R3–6 Reelin Fragment

Reelin acts as a positional cue for dentate granule cells during development, since rescue of granule cell lamination in Reelin-deficient *reeler* mice could be achieved when Reelin was present in normotopic position, provided by a wild-type co-culture (Zhao et al., 2004). Conversely, infusion of Reelin-blocking antibodies (CR-50) into the hippocampus of normal adult mice induced GCD locally (Heinrich et al., 2006). These findings established a role for Reelin in stabilizing the lamination of the dentate gyrus. Accordingly, we hypothesized that the KA-triggered motility of dentate granule cells toward the Reelin-free hilar area might be caused by a loss of the positional cue. Granule cells constitutively express the Reelin receptors ApoER2 and VLDLR (Haas et al., 2002) and have been shown to maintain their expression after KA injection (Müller et al., 2009). Here, we demonstrated that addition of recombinant R3–6 Reelin fragment (1 nM) was able to prevent the movement of granule cells observed after KA application alone. The R3–6 Reelin fragment has been shown to be sufficient for activating the Reelin signal transduction cascade on target cells (Jossin et al., 2004). Despite the existence of several Reelin isoforms *in vivo*, only fragments containing R3–6 are capable of binding ApoER2 and VLDLR, and both receptors alone are capable of binding Reelin with similar affinity. The residues Lys-2360 and Lys-2467, found in Reelin R3–6, are directly responsible for coordinated binding of Reelin to the conserved ligand binding domains of ApoER2/VLDLR (Yasui et al., 2007, 2010). In agreement with these findings, application of Reelin fragments containing R5–6 to *reeler* cortical explants is sufficient to induce Dab1 phosphorylation and to normalize cortical lamination (Jossin et al., 2004).

So far, our findings point to a stop signal function of Reelin, which might be lost in the presence of KA. In contrast, a recent study did not detect the formation of GCD after conditional Reelin knockout in the adult dentate gyrus (Lane-Donovan et al., 2015). In these mice, however, Reelin was not ablated completely; they still exhibited around 5% of the initial Reelin concentration. Since very low Reelin concentrations (1 nM) were sufficient to obtain significant effects in our hands and also in other studies (Leemhuis et al., 2010), the incomplete Reelin knockout may explain the controversy.

Thus, our results indicate that the central Reelin fragment is able to prevent the KA-mediated migration of eGFP-positive granule cells and support the role for Reelin as a positional signal for granule cells in the adult hippocampus.

## Author Contributions

CO: performed experiments, data analysis, manuscript writing; GM: performed experiments, data analysis; JG: performed experiments, data analysis; AK: data analysis and Matlab^®^ calculations; MF: confocal imaging and data analysis; UE: data analysis; CH: conception, supervision, manuscript writing.

## Conflict of Interest Statement

The authors declare that the research was conducted in the absence of any commercial or financial relationships that could be construed as a potential conflict of interest.
