# Supplementary figures and images for: Seizure-Induced Motility of Differentiated Dentate Granule Cells Is Prevented by the Central Reelin Fragment
Source: Front Cell Neurosci. 2016 Jul 28;10:183. doi: 10.3389/fncel.2016.00183 (PMC4963407; doi:10.3389/fncel.2016.00183)

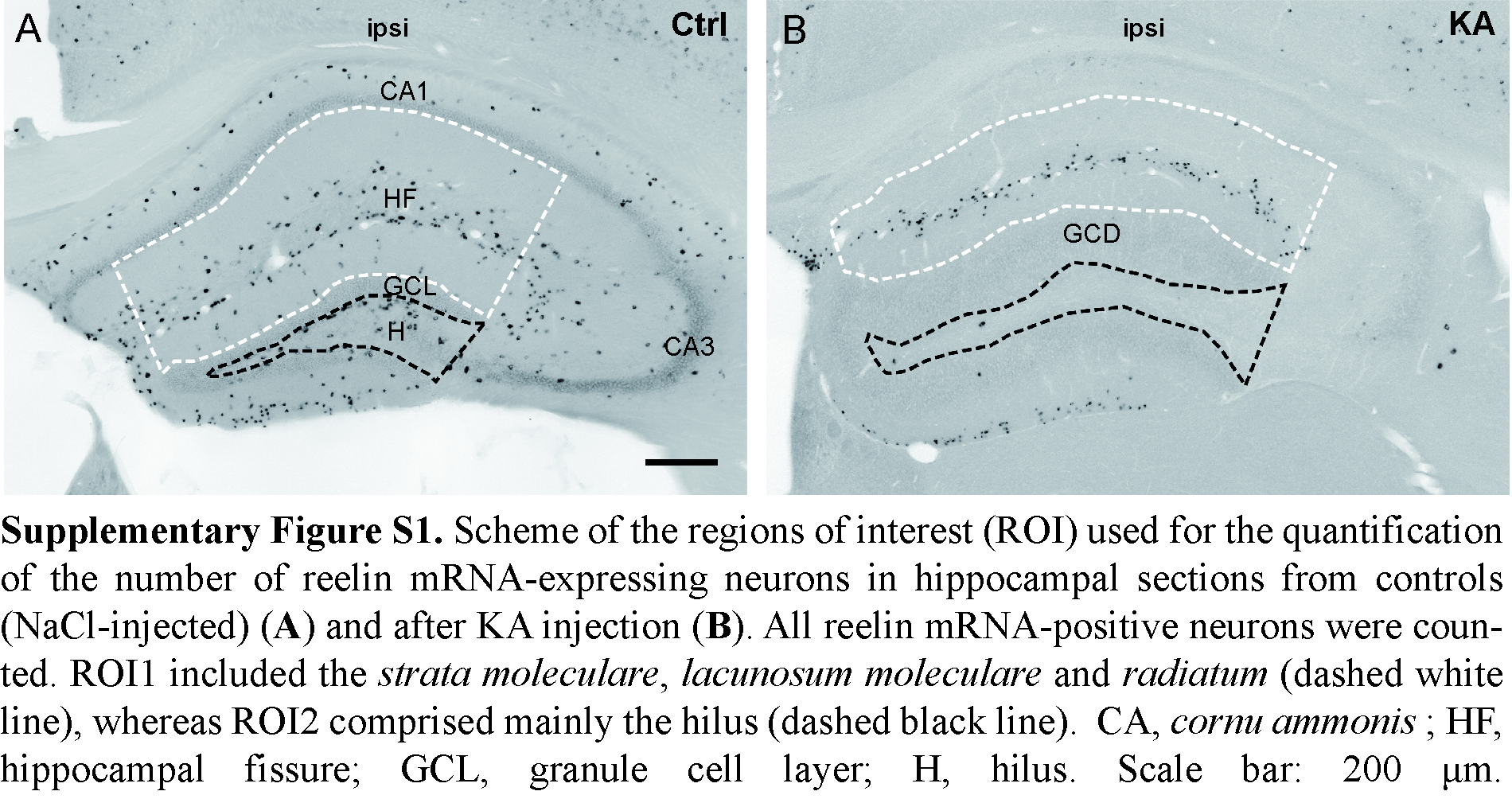

Supplement: Supplementary file 1 [file Image_1.TIF]

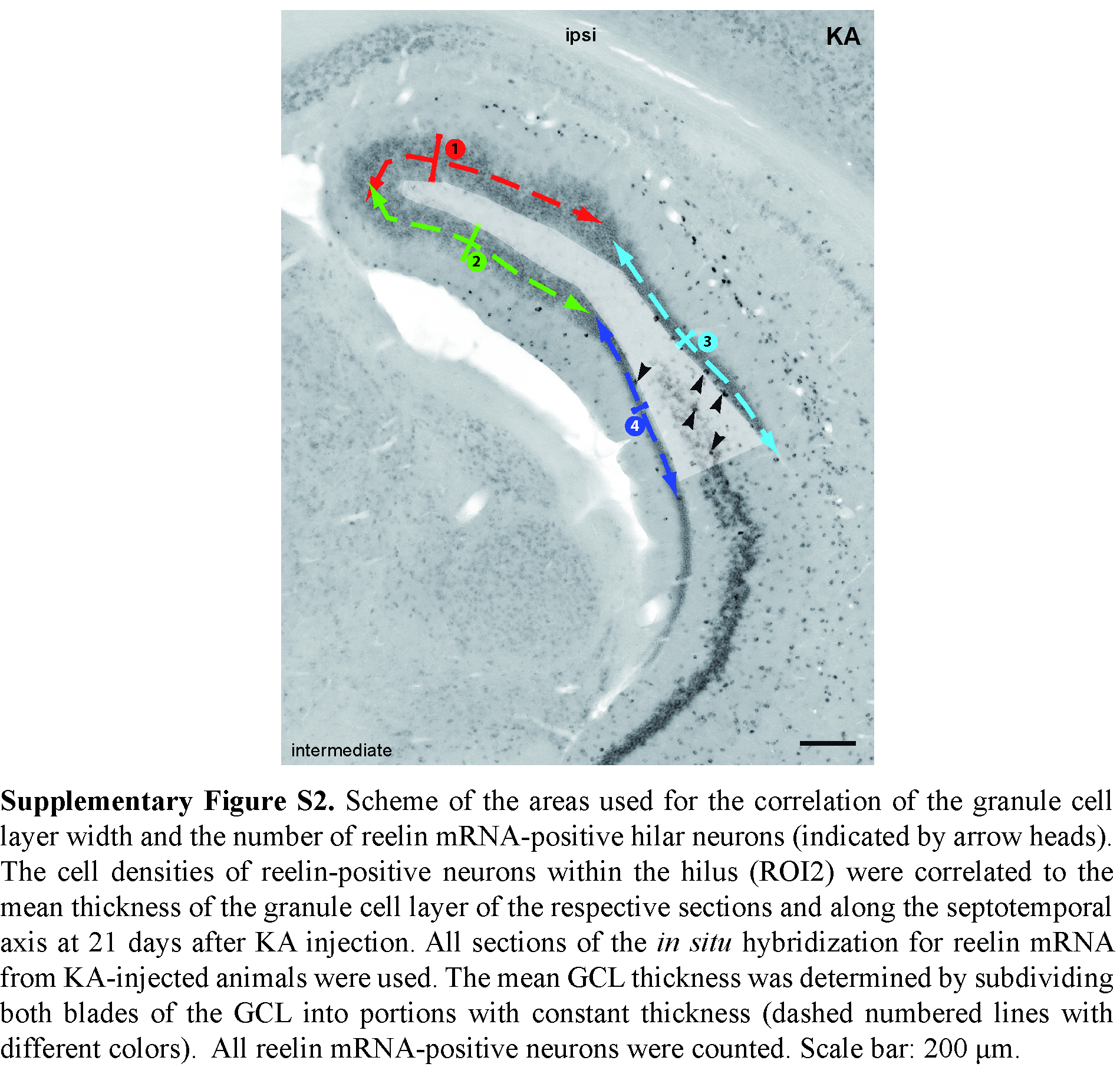

Supplement: Supplementary file 2 [file Image_2.TIF]

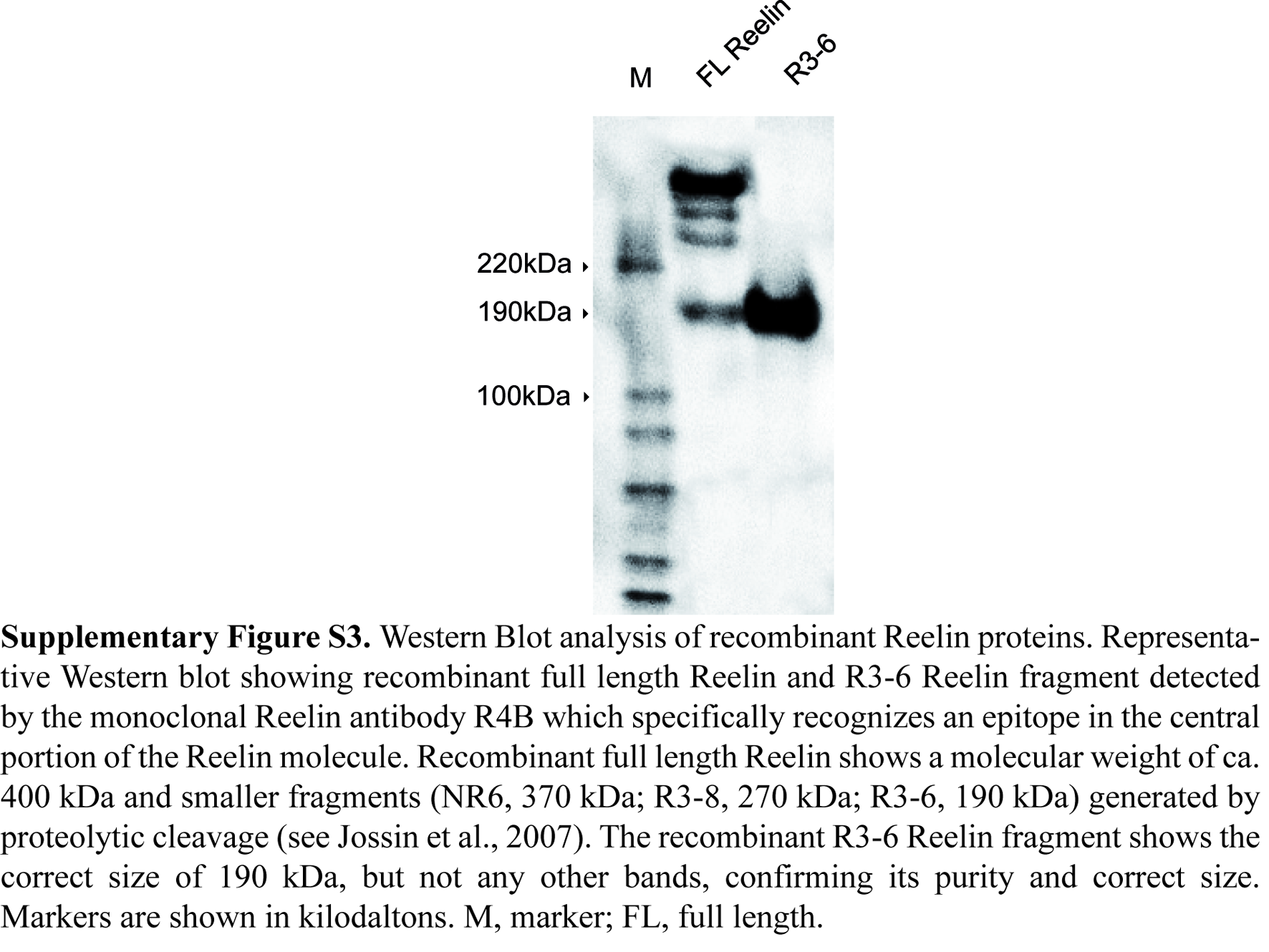

Supplement: Supplementary file 3 [file Image_3.TIF]

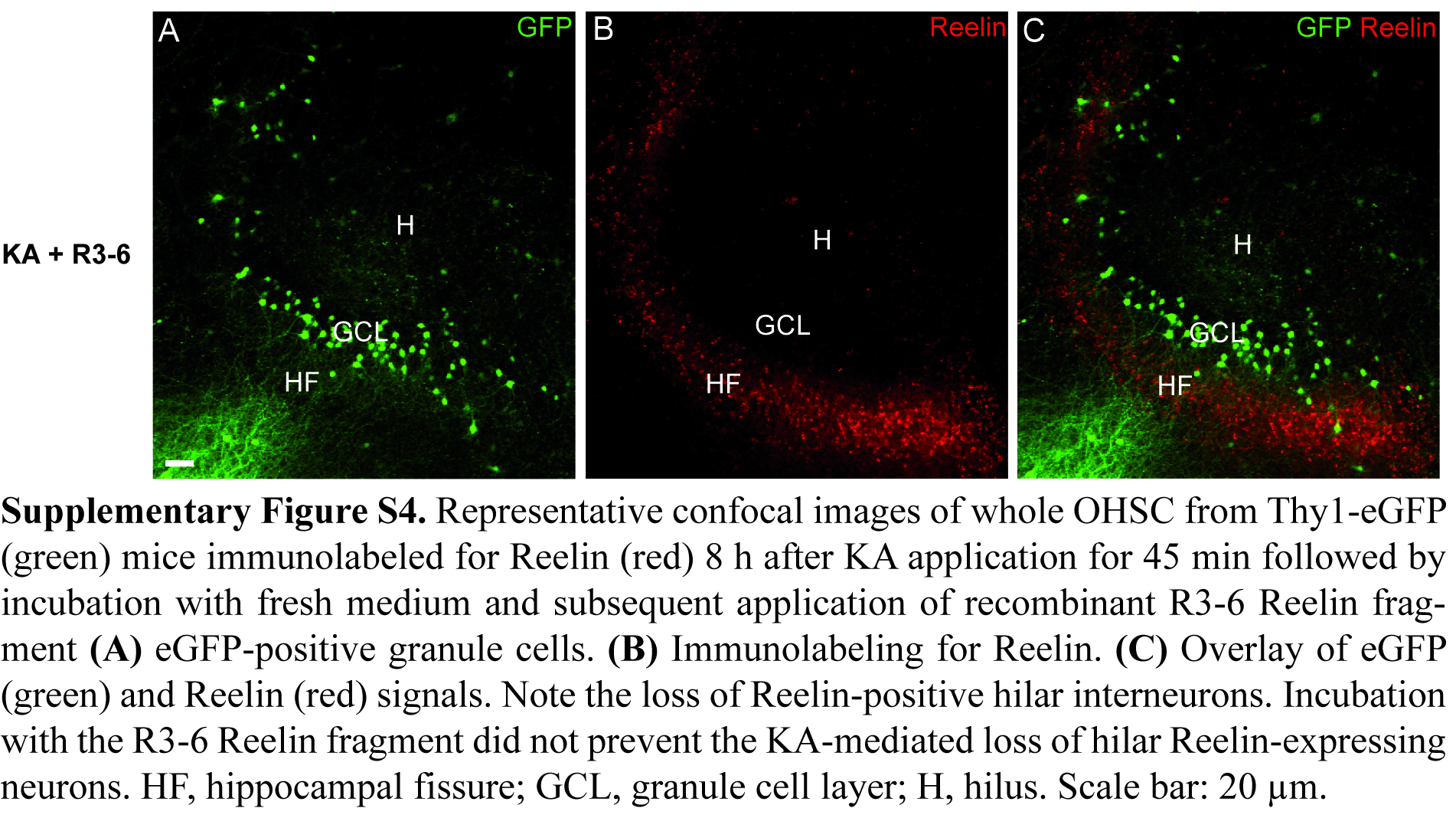

Supplement: Supplementary file 4 [file Image_4.TIF]

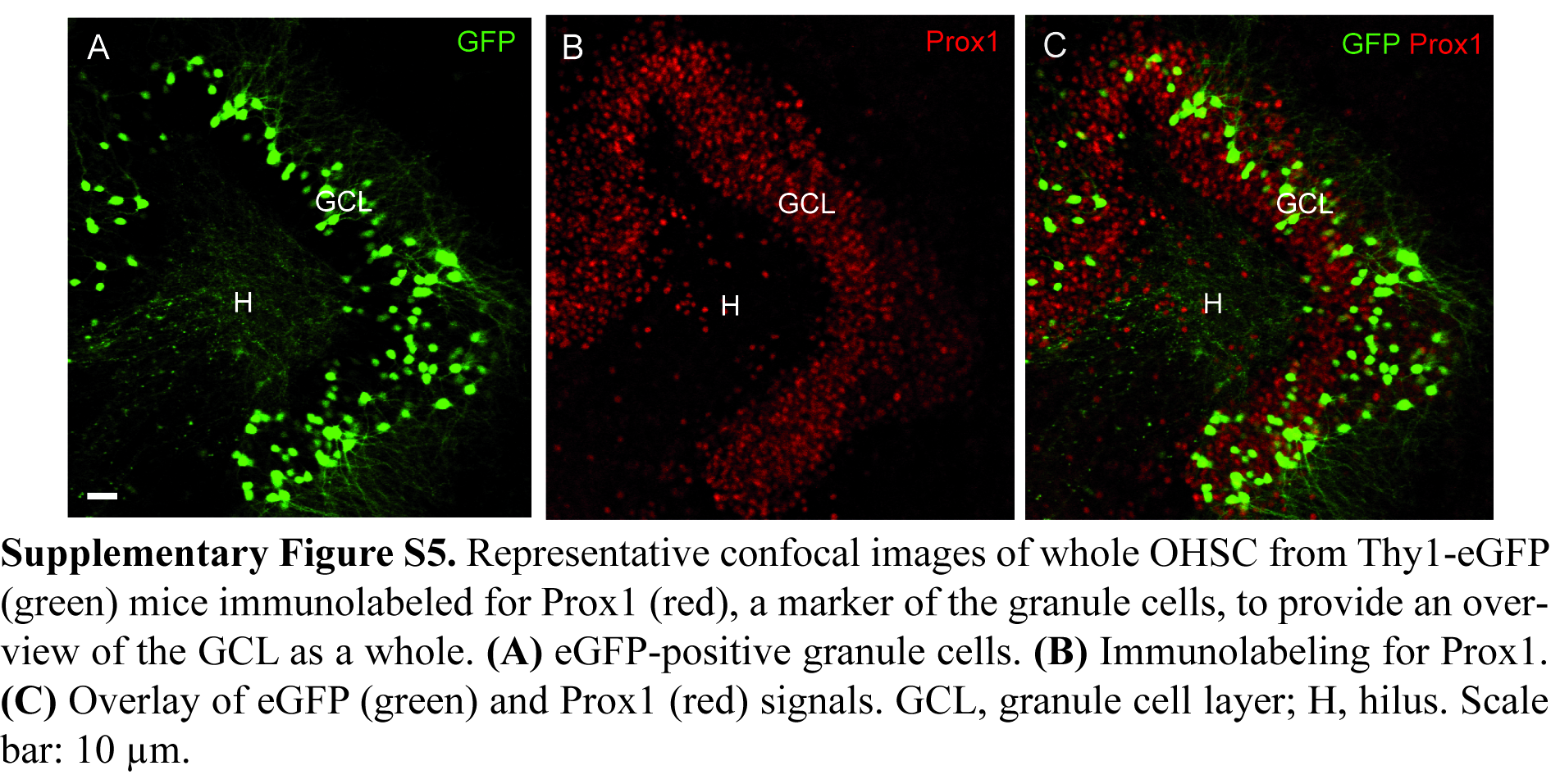

Supplement: Supplementary file 5 [file Image_5.TIF]
